# Cloning and molecular characterization of *TaERF6*, a gene encoding a bread wheat ethylene response factor

**DOI:** 10.22099/mbrc.2018.30339.1336

**Published:** 2018-12

**Authors:** Shahrzad Haghir, Abbas Alemzadeh

**Affiliations:** Department of Crop Production and Plant Breeding, School of Agriculture, Shiraz University, Shiraz, Iran

**Keywords:** Hydrophobic cluster analysis, Phosphorylation sites, Phylogenetic, Poaceae

## Abstract

Ethylene response factor proteins are important for regulating gene expression under different stresses. Different isoforms for ERF have previously isolated from bread wheat (*Triticum aestivum* L.) and related genera and called from *TaERF1* to *TaERF5*. We isolated, cloned and molecular characterized a novel one based on *TdERF1*, an isoform in durum wheat (*Triticum turgidum* L.) and called *TaERF6*. Its cDNA was synthesized, sequenced and compared with genomic sequence to figure out intron and exon regions and determine coding sequence region. The length of *TdERF1* gene was 1939 bp and cDNA was 1065 bp including two exons, the first one 259 bp and the second one 806 bp separated by a 874 bp intron with a 111 bp 5'-UTR (untranslated region) and 401 bp 3'-UTR. *TaERF6* encodes a 353 amino acids protein with nearly 99% identity to TdERF1. Hydrophobic cluster analysis revealed an N-terminal hydrophobic domain contains a highly conserved motif with the consensus sequence of M [C/L/Y] [G/R] [G/R/P] [A/G/V/L/R] [I/L/R/S/P/Q] [L/I/R/H] and hydrophobic clusters in AP2/ERF domain of which tends to form -sheet. Three monopartite nuclear localization signals also identified in TaERF6 that play important role in getting back into the nucleus. The results showed several putative phosphorylation sites in TaERF6 that a motif from residues 246 to 266, the CMVII-4 motif, was predicted to phosphorylate by different kinase proteins and play important roles in TaERF6 function. Phylogenetic analysis showed 7 clusters (I to VII) and 10 subclusters according to their relatedness in Poaceae family.

## INTRODUCTION

Biotic and abiotic stress stresses such as salinity, drought, extreme temperatures, heavy metals, insects and diseases have adverse impacts on the growth and production of plants [1, 2]. To grow and thrive under adverse environmental conditions plants have to use especial proteins to regulate responses to stress signals [3, 4]. Some proteins like transporters or exchangers are directly involved in regulating toxic ions loading into xylem tissue while some proteins regulate these processes [5]. The most important proteins in the regulation of stress responses are transcription factors of which play hub roles in gene expression of target genes via specific binding to *cis*-elements in promoter regions [6]. 

One of the most important superfamily of transcription factors in stress response mechanisms is AP2/ERF superfamily that grouped into five families including ERF (ethylene responsive factor), AP2 (APETALA2), DREB (dehydration-responsive element binding protein), RAV and others [7]. Among these families, ERF family members are important group of them with only one conserved sequence (AP2 domain) related to this superfamily, which has drawn more attention in recent decades [6, 8]. It has been previously shown that different members of this family are involved in various biotic and abiotic stress responses and/or involved in signaling pathways and have hub roles in regulatory networks [8, 9]. Many stress-inducible ERF family members have been isolated and characterized and shown they are major transcription factors involved in biotic and abiotic stress responses via regulating various genes expression through binding to *cis* elements in the promoter regions [10].

Characterization of a protein molecule and compare to other proteins in the same family may be useful way to identify differences and similarities between different isoforms and figure out the differences in their functions [11]. Nowadays, significant amount of data are available in databases such as National Center of Biotechnology Information (NCBI) or (Expert Protein Analysis System) ExPASy of which allowing a comparison of different proteins within and between genera [12]. Hence, the bioinformatic field is an emerging and rapidly developing way which allows people to analyze biological data and comparison them with others [11]. Different aspects of proteins can determine by bioinformatic tools and figure out the role of the protein in various cellular processes. Some modifications in the protein structure such as phosphorylation that is catalyzed by kinases have important roles in the function of protein. Today, we know some amino acids, serine, threonine and tyrosine have key roles in this process and protein phosphorylation is done at them [13] and we are able to predict the phosphorylation sites that possibly regulated by phosphorylation. 

Different isoforms of ERF proteins in wheat have previously reported and called TaERF1 to TaERF5, in this study we successfully isolated a new isoform based on *TdERF1* sequence, an ortholog from *Triticum turgidum*, and calle *TaERF6*. TaERF6 was characterized using different bioinformatic programs and compared with other isoforms in wheat or other genera of Poaceae family through phylogenetic tree. We also showed evolutionary relationship between different isoforms and revealed differences and similarities of sequences within and between clusters and subclusters.

## MATERIALS AND METHODS

**Plant materials and cultivation methods:** Seeds of bread wheat were surfaced sterilized for 7 min in ethanol (70%) and washed five times with distilled water and then planted in plastic pots with a soil mixture consisting sand and grit (1:1, v/v) under glasshouse conditions (16 h of light and 25/16º C day/night temperature). Leaves of bread wheat plants were harvested and frozen in liquid nitrogen and then kept at -80°C until used.

**Primer design:** A primer pair based on upstream and downstream sequences of *TdERF1* was designed to amplify its orthologs from *Triticum aestivum* and paraloges from *Triticum turgidum* genomes. The sequences of primers were: ERF1P1F: 5'-CAT TCT TCT TTC TTT TGT TTC GC-3', and ERF1P1R: 5'-CAG GAA TAC AAA ATA AGA GTT CC-3'. 

**DNA and RNA extraction and synthesis cDNA:** The genomic DNA of samples was extracted from leaves using CTAB method [14]. The RNA from leaves were extracted using RNA Extraction Kit (Denazist, Iran) and immediately treated with RNase-free DNase I (Fermentas, Germany) to remove DNA contamination. The quality of extracted DNA and RNA were assessed by electrophoresis on 2% agarose gel. First-strand cDNA was synthesized from extracted RNA by RevertAid M-MuLV Reverse Transcriptase (Fermentas, Germany), with oligo (dT) primer [15]. 

**PCR Reactions and cloning of PCR products:** PCR reactions were performed in a final vlume of 20 μl reaction containing 50 mM KCl, 10 mM Tris-HCl (pH 8.3), 1.5 mM MgCl2, 0.3 μM each primer, 200 μM dNTP, 1 unit of Taq DNA polymerase and 1 μg genomic DNA as template. The PCR reactions were done under following conditions: 4 min at 96°C, 40 cycles at 95°C for 45 s, 59°C for 30 s, and 72°C for 3 min, with a final extension for 10 min at 72°C and then PCR product was separated on 2% agarose gel [16]. The amplified product was cloned into the pTZ57R/T vector using the TA cloning kit (Fermentas, Germany) and positive clones were selected and sequences [17].

**Bioinformatics analysis:** The homology comparison of DNA sequences and deduced amino acid sequences were carried out by ClastalW program (http://www.genome.jp/tools-bin/clustalw). The percentage of amino acid identity was determined by Clustal Omega program (http://www.uniprot.org/align/) or Kalign program (https://www.ebi.ac.uk/Tools/msa/kalign/). The database screening was performed using BLAST program at the National Center for Biotechnology Information, NCBI (https://blast.ncbi.nlm.nih.gov/Blast.cgi) and ExPASy (https://expasy.org/proteomics). Protein domain prediction was performed by RPS-BLAST using NCBI Conserved Domains Database (CDD) (https://www.ncbi.nlm.nih.gov/Structure/ cdd/wrpsb.cgi) and OMA (https://omabrowser.org/oma/home/). The functional information on proteins was obtained using UniProt Knowledgebase (UniProtKB) (http://www.Uniprot.org/ uniprot/). The nucleotide sequences were translated to amino acid sequences using translate tool on the SIB Bioinformatics Resource Porta, ExPASy (http://web.expasy.org/ translate/). Hydrophobic cluster analysis (HCA) was carried out using the web-based interface, HCA 1.0.2 program, at http://mobyle.rpbs.univ-paris-diderot.fr/cgi-bin/portal.py?form=HCA#welcome. Strong hydrophobic amino acids [isoleucine (I); leucine (L); valine (V); phenylalanine (F)] and moderately hydrophobic amino acids [tyrosine (Y); methionine (M); tryptophan (W)] separated by at least four nonhydrophobic residues, or by a proline, were located into distinct clusters, which were grouped by outlines. NLSs (Nuclear Localization Signals) were predicted by cNLS mapper program (http://nls-mapper.iab.keio.ac.jp/cgi-bin/NLS_Mapperform.cgi). The phosphorylation sites were predicted using NetPhos 3.1 (http://www.cbs.dtu.dk/services/ NetPhos/). The kinase specific eukaryotic protein phosphorylation sites were predicted by GPS 3.0 software [18]. The molecular weight was computed by compute PI/MW tool, ExPASy (http://web.expasy.org/compute_pi/). The selected sequences were used to draw an unrooted phylogenetic tree by MEGA6 software [19].

## RESULTS

We assumed that orthologs between *Triticum turgidum* L. and *Triticum aestivum* L. may play similar roles in plant responses to salt and drought stresses. Hence, we used the cDNA sequence of *TdERF1*, a gene encoding ERF protein in *T*.* turgidum*, to search for paralogs and orthologs of that in *T*.* turgidum* and *T*.* aestivum*, respectively, using NCBI (National Center for Biotechnology Information) blast program. We found five sequences (accession numbers: KJ689809.1, KJ689810.1, KJ689811.1, KJ689812.1 and KJ689813.1) (Supplementary Data 1) in *T*.* turgidum* and 9 sequences (accession numbers: AK335399.1, AK452621.1, AY781361.1, AF542184.1, AK332004.1, AY781352.1, AY271985.1, AY271984.1, AJ515477.2 ) in *T*.* aestivum* (Supplementary Data 1). Sequences analysis showed that the sequences of KJ689812.1 and KJ689813.1 from *T*.* turgidum* are exactly the same, and, in *T*.* aestivum*, the sequences of AK335399.1 and AK452621.1 and the sequences of AY781352.1 and AK332004.1 are the same, therefore we used only one of them in later analysis. The sequence of AY781361.1 belonged to DREB family, hence, it was removed. Finally, six genes (*TaERF1*, *TaERF2*, *TaERF3*, *TaERF4*, *TaERF5* and *TaERF6*) were selected from *T. aestivum*.

**Figure 1:**
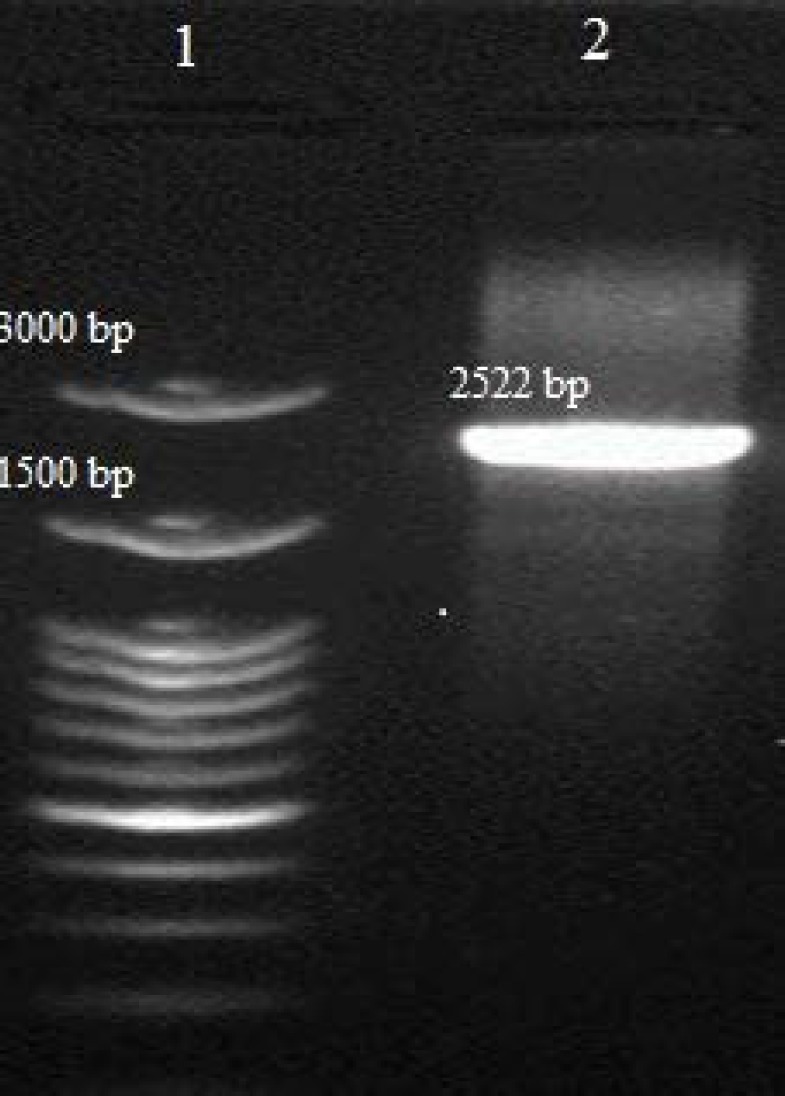
Amplification of *TaERF6* from bread wheat genome using ERF1P1F and ERF1P1R. lane 1) DNA size marker, 2) A 2522 amplified fragment including *TaERF6*.

**Figure 2:**
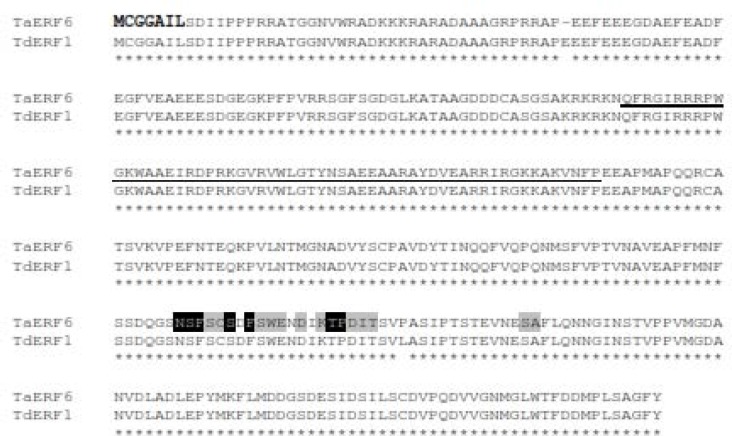
The deduced amino acid sequence of TaERF6 and comparison with TdERF1. AP2/ERF domain stretches from residue 110 to 167 that is underlined. N-terminal motif of MCGGAIL is shown in bold. CMVII-4, a predicted phosphorylated motif, is shown black and gray shading indicates identical and conserved amino acid residues in this motif.

TdERF1 from T. turgidum had 99% similarity to the sequence of AK335399.1 in T. aestivum and it was hypothesized that this sequence is TdERF1 that transferred from T. turgidum to bread wheat genome. A primer pair based on upstream and downstream sequences of TdERF1 was designed to amplify the ortholog of this gene from bread wheat genome. A 2522 bp fragment was amplified during PCR with bread wheat genome as template (Fig. 1); the result of sequencing confirmed the hypothesis and the gene called TaERF6. A 1062 bp fragment was also amplified when cDNAs prepared from RNA extracted from leaves used as template in PCR reaction with the same primer and its sequence was determined. As expected, the sequence was related to a gene encoding an ERF protein. TaERF6 is predicted to encode a 353 amino acids protein with nearly 99% identity to TdERF1 (Fig. 2). Comparison of the nucleotide sequences of upstream (5’-UTR) and downsream (3’-UTR) regions of these genes showed that upstream regions were 100% identical while downstream regions were only around 84% identical. The predicted molecular weight and isoelectric point of TaERF6 are 38.7 kDa and 4.7, respectively.

**Figure 3:**
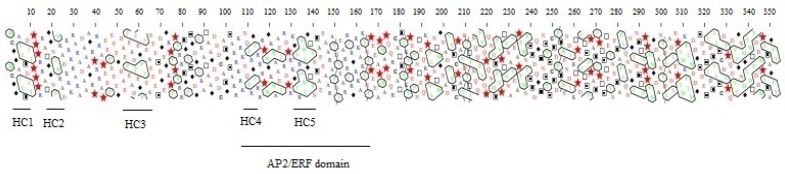
Hydrophobic cluster analysis (HCA) of TaERF6. Each hydrophobic cluster is shown by HCA. Singlet amino acid (one isolated hydrophobic amino acid) is circled. Classic symbols were used for amino acids except for proline ( * ), glycine ( ● ), theronine ( ▲ ) and serine ( ◄ ).

To characterize the structure of TaERF6, its sequence was analyzed by hydrophobic cluster analysis. The results showed that TaERF6 has a modular structure (Fig. 3). The N-terminal of this protein includes a hydrophobic domain contains a highly conserved motif (MCGGAIL). A number of singlet amino acids (S) (one isolated hydrophobic amino acid) separated by proline are located in residues 72-84. The AP2/ERF domain can be divided into two parts; the first part is a region contains hydrophobic clusters of which tends to form -sheet and the second part contains a number of singlet amino acids stretch from residues 150 to 169. Another region stretches from residues 191 to 239 of which tends to form -helix. The C-terminal of TaERF6 also contains hydrophobic clusters disperse in this region (Fig. 3). Polar amino acids, arginine (R) and lysine (K), are observed at N-terminal and central regions of this protein and they are absent in C-terminal region.

PredictNLS predicted three monopartite NLS; one of them was within the first 34 amino acids of TaERF6, and two other signals are located in residue 101 and 103 (Table 1). All of these NLSs belong to class 1 of NLSs. 

**Table 1:** Nuclear localization signals prediction for TaERF6

**Putative NLS**	**Model**	**Score**	**position**
RADKKKRARAD	[K/R]X_2_[K/R]_4_X[K/R]	5.5	24 - 34
GSAKRKRKNQ	X[K/R]_5_X	5	101 - 110
AKRKRKNQFR	X[K/R]_5_X	7	103 - 112

Various features of proteins are regulated via phosphorylation of target proteins by protein kinases at specific amino acid residues, threonine, serine and tyrosine [11]. Hence, it may useful to identify sites with potential for phosphorylation. The results revealed several putative phosphorylation sites in TaERF6 that the sites with a strong prediction value above 0.9 were considered (Table 2). It was predicted that a motif from residues 246 to 266, the CMVII-4 motif, is phosphorylated by different kinase proteins. 

**Table 2:** Predicted phosphorylation sites in TaERF6

**Position**	**NetPhos Score**	**Amino acid**	**Kinase protein**	**sequence**
**18**	0.955	T	AGC/RSK/MSK/RPS6KA5	PPRRA**T**GGNVW
**69**	0.989	S	CMGC/CK2	EAEEE**S**DGEGK
**81**	0.988	S	NEK/NEK11	FPVRR**S**GFSGD
**84**	0.973	S	TKL/STKR	RRSGF**S**GDGLK
**102**	0.964	S	AGC/PKC/PKCa/PRKCB	DCASG**S**AKRKR
**142**	0.925	S	CMGC/CK2	LGTYN**S**AEEAA
**181**	0.968	S	CMGC/CDK/CDK4	QRCAT**S**VKVPE
**204**	0.912	Y	TK/Eph/EphB1	GNADV**Y**SCPAV
**247**	0.969	S	CAMK/MAPKAPK/MNK/MNK2	DQGSN**S**FSCSD
**249**	0.979	S	PDHK/PDK2	GSNSF**S**CSDFS
**254**	0.993	S	STE/STE20/PAKA/CLA4	SCSDF**S**WENDI
**274**	0.991	S	PLK	ASIPT**S**TEVNE
**318**	0931	S	CMGC/CK2	LMDDG**S**DESID

**Figure 4:**
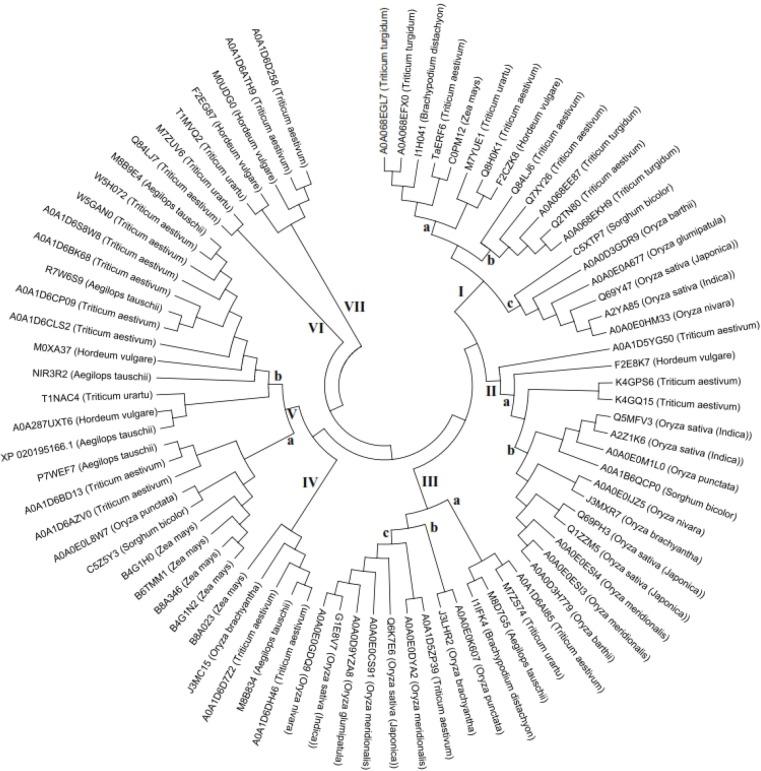
An unrooted phylogenetic tree of Poaceae family ERF proteins. The full length deduced amino acid sequences of ERF proteins were aligned by ClustalW and phylogenetic tree was drawn using maximum likelihood method.

The homology search for the amino acid sequence of TaERF6 using ExPASy blast program found 350 sequences that after removing identical sequences, 236 sequences obtained that only 82 sequences were related to Poaceae family* (Supplementary Data 2). *All selected sequences have only one conserved sequence (AP2 domain) related to AP2/ERF superfamily (accession number: cl00033) that indicated they belong to ERF family. These 81 sequences were used to draw an unrooted phylogenetic tree (Fig. 4). The results of phylogenetic tree showed 7 clusters (I to VII) and 10 subclusters according to their relatedness. Cluster I with 19 members grouped into three subgroups, Ia (8 members), Ib (5 members) and Ic (6 members) (Fig. 4) that all sequences of this cluster have N-terminal motif of MCGGAIL. All sequences of cluster IIa also have N-terminal motif of MCGGAIL, while all members of cluster IIb have N-terminal motif of MCGGAII except for A0A1B6QCP0 (*Sorghum bicolor) that isoleucine 7 was substituted with a leucine residue.*

In cluster III, all members of subclusters IIIb and IIIc have N-terminal motif of MCGGAII/L, but the members of subcluster IIIa are different in this sequence and only one of them, I1IFK4 (*Brachypodium distachyon)*, has MCGGAIL sequence and the other members which related to *Triticum* or *Aegilops* genera have different sequences. Two members of this subcluster, A0A1D6AI85 (Triticum aestivum*) *and M7ZS74 (Triticum urartu*), have the same MYGGASL motif, of which appears only in them and some members of cluster V while other ERF sequences were studied in this study have not this motif. All members of cluster IV have *N-terminal motif of MCGGAIP/L, but the members of cluster V are different in the sequence of this motif. The members of subcluster Va* may be devided into 2 subgroup, one group have *N-terminal motif of MYGGA/VSL and the other group have MCGGAIL motif; and finally, the sequences fell into clusters VI and VII are different regarding this motif. 

All sequences contained only a conserved AP2/ERF DNA binding domain consists 58 amino acid residues. Twenty-five residues in this sequence were not variable among different sequences including: R_6_, R_8_, W_13_, A_14_, I_17_, R_18_, D_19_, P_20_, K_22_, G_23_, R_25_, W_27_, L_28_, G_29_, T_30_, A_37_, A_38_, R_39_, A_40_, Y_41_, A_45_, I_48_, G_50_, K_52_ and A_53_. Only the members of three clusters, IIa, IIb and IIIb have exactly the same sequence, and in other groups, the members of the same cluster is not exactly the same regarding this domain and are different at least in one residue.

## Discussion

We have been interested in genes encoding proteins are involved in abiotic stresses. ERF is one the most important proteins in abiotic stress responses. Wheat is an important crop in the world, but there are few studies in which its genes have been characterized. We cloned and characterized *TaERF6*, the gene encoding an ERF protein in bread wheat. 

Although, there are 5 isoforms with high similarity to *TdERF1* in *T. turgidum*, we used only *TdERF1* to figure out its paraloges in *T. aestivum* since it has been reported that *TdERF1* plays important roles in drought and salt stresses [20]. A 2451 bp fragment was amplified when *TdERF1* used as target DNA in the PCR reaction from *T. turgidum* genome with ERF1P1F and ERF1P1R primers (Fig. 1), while when the genome of *T. aestivum* was used as template, a 2522 bp fragment was amplified with these primers. The difference may relate to its coding region, introns, upstream and/or downstream regions. The length of *TdERF1* gene is 1939 bp and cDNA is 1065 bp (the coding sequence consists of two exons, the first one 259 bp and the second one 806 bp separated by a 874 bp intron) with a 111 bp 5'-UTR (untranslated region) and 401 bp 3'-UTR, while the length of *TaERF6* gene is 1936 bp and cDNA is 1062 bp (the coding sequence consists of two exons, the first one 256 bp and the second one 806 bp separated by a 874 bp intron) with a 112 bp 5'-UTR and 474 bp 3'-UTR. It revealed that most difference between these genes is related to 3'-UTR region. The sequence of *TaERF6* was not identical to other sequences of ERF genes in bread wheat indicating that this is a new isoform. As results showed, the sequences of *TaERF6* and *TdERF1* are very similar to each other indicating these genes have a common ancestor and this gene was originally transferred from tetraploid wheat to hexaploid wheat. The sequence search of *TaERF6* revealed that the gene located in sub-genome A confirming this gene transferred from *T. turgidum* genome (AABB). The results indicated that various isoforms can be distinct from each other in PCR reaction by designing specific primers for their 3'-UTR regions. It has been previously reported that different isoforms can be distinguished when the 3’-UTR region is selected as target DNA in PCR reaction [11, 21]. 

The HCA is based on the hypothesis that hydrophobic amino acid residues are not randomly distributed while tend to form clusters and its representation actually fits very well with fundamental principles that govern protein folding, indicates critical information hidden in primary sequences, and offers a useful way to cope with limitations of one-dimensional methods [22]. The HCA analysis of N-terminal region from TaERF6 showed two hydrophobic clusters (HC1 and HC2) that HC1 separated from HC2 by proline residues (Fig. 3). Proline often breaks secondary structures and it is considered as a cluster breaker [22]. The proline breaks the H-binding pattern that leads in a break in -helix and terminates the domain. In the N-terminal of TaERF6, a 110011 cluster (P code: 51 and Q code VUV) [22] is separated from the next hydrophobic cluster by proline residues; this cluster displaying a preference to be associated with -helix [23]. The highly conserved motif, MCGGAIL is located in HC1 cluster. It has been shown that this motif makes a limitation for interaction between ERF proteins and the protein kinase and deletion of this motif increased the interaction of ERF proteins with map kinase [24]. A number of singlet hydrophobic amino acids found in two parts of TaERF6; these single amino acids may play a role in the interaction between proteins [25]. 

Due to the existence of numerous hydrophobic clusters in the C-terminal of TaERF6, this region can be considered as a region for trans-activation process. It has been shown that the presence of numerous hydrophobic amino acids in the C-terminal mediates trans-activation process [26]. The above suggested that TaERF1 was a novel member of the ERF family and might be involved in the modulation of gene expression and interaction with other proteins in the nucleus.

Nucleus is the most important compartment in the cell, where various essential processes take place. After synthesis, proteins are exported from the nucleus and in some cases, after manufacturing they must get back into the nucleus to do their duties. Some sites in the protein sequence are necessary for this import, called nuclear localization signals, NLSs.

To identify any NLS in the sequence of TaERF6, an analysis tool, PredictNLS which identifies NLSs in the query sequence using a database of experimentally deposited NLS motifs, was used. It is expected that the prediction of PredictNLS has less false positives, since this tool works based on experimental data [27]. Kosugi et al. (2009) divided NLSs into six classes and based on their classification, all NCLs in the sequence of TaERF6 fall into class 1, the members of this class have at least four consecutive basic residues. It has been shown that classical NCLs may play important roles in transporting proteins into the nucleus since 45% of protein molecules in yeast have these sequences to enter the nucleus [29]. The results indicated that TaERF6 has the potential to enter the nucleus through the classical nuclear import pathway. These results may imply that TaERF6 gets back into the nucleus to bind to specific *cis* elements in the promoter regions of some genes.

Interaction between ERF and other proteins has been reported in different surveys. Protein phosphorylation at serine, threonine and/or tyrosine residues may influence different features of a protein such as interaction with other proteins or binding to *cis* elements [30]. It was reported that ERF proteins are phosphorylated by different mitogen-activated protein kinases that plays important role in the regulation of these proteins [31]. It was shown that CMVI-3, CMVII-4, CMIX-5 and 6 motifs of ERF proteins are putative sites for phosphorylation by map kinases [32]. The CMVII-4 motif was found in ERF sequence stretches from residues 246 to 266 (Fig. 2) which was predicted to be phosphorylated by CAMK/MAPKAPK/MNK/MNK2/PDHK/ PDK2/STE/STE20/PAKA/CLA4 at S 247, 249 and 254 residues. CAMK are Ca^2+^/ calmodulin-dependent protein kinases that are activated by increases in the concentration of intracellular Ca^2+^, and as known, calcium ions elevation activates stress signaling [33]. Map kinases are recognized as critical proteins in different cellular events in plants. These proteins are known play important roles in stress responses [34, 35]. TaERF6 was predicted to be phosphorylated by MNK proteins, and especially MNK2 which phosphorylates and regulates protein synthesis under stress conditions [36], at the serine 247 residue. MNK proteins are phosphorylated by P38MAPK [36] which are a class of MAP kinase proteins that involved in abiotic stress response mechanisms [37]. Based on previous reports, these kinase proteins involved in gene expression and regulation of various protein activities under stress conditions, and to our knowledge, there is no report to indicate ERF proteins in wheat are phosphorylated and regulated by these kind enzymes. All to all, this information may indicate that TaERF6 is an important regulatory protein in abiotic stresses.

Previous phylogenetic study revealed 8 groups and 11 subclusters for ERF family from different plant species according their relationships [38]. In this study, the ERF proteins from Poaceae family were grouped into 7 clusters and 10 subclusters. All members of both clusters I and IIa, have N-terminal motif of MCGGAIL, while in all members of cluster IIb, *leucine 7 was substituted with an isoleucine residue *except for one isoform from *S. bicolor. Both of these amino acids are aliphatic, nonpolar and neutral at pH 7 which have nearly the same role in the function of proteins. As it has been shown, this motif has an important role in the interaction of ERF protein with other proteins *[24], probably, all sequences in cluster I and II have the same role in interaction with other proteins and target DNA recognition. *All members of subgroup IIb belonging to *Oryza* genus contains *MCGGAII motif except one of them that comes from *Sorghum *genus that has MCGGAIL. In addition, two others isoforms from *S. bicolor* of which were located in cluster Ic (C5XTP7) and Va (C5Z5Y3) have the same motif MCGGAIL in N-terminus. It may suggest this change has occurred in ERF protein after the separation of *Oryza* and *Sorghum* genera in Poaceae family. In some genera such as *Zea*, most isoforms were located in a cluster and subcluster, subcluster Va, while in most genera, different isoforms were dispersed among different clusters, it means, after separation of genera, in some of them, the isoforms have been widely undergone evolutionary changes, while in others, such as *Zea*, the changes were less. 

It has previously reported that ERF proteins have a highly conserved N-terminal motif (MCGGAII/L) [39], but in this study we found that only residues 1, 3 and 4 are highly conserved while other residues in this motif are more variable and residues 5 and 6 are the most variable ones. Finally, it can be suggested the consensus sequence M [C/L/Y] [G/R] [G/R/P] [A/G/V/L/R] [I/L/R/S/P/Q] [L/I/R/H] for this N-terminal motif. Residues 3 and 4 in most sequences are G and one or a few sequences have other amino acids in these positions. Residue 2, in most sequences is C and in a few sequences was substituted with a tyrosine residue; only in one sequence from Aegilops tauschii related to cluster IIIa was substituted with a leucine residue. The sulphur atoms of two cysteines linked to each other and form a disulfide bound, which serves an important structural role in the protein.

Another important sequence in ERF proteins is AP2/ERF DNA binding domain, which has critical role in binding to *cis* elements [40]. The invariable residues present in this domain probably plays important role in the function of the domain. In all sequences the three last residues are NFP except for an isoform from *T. aestivum* (Q84LJ6) belongs to subcluster Ib. This sequence belongs to computer-annotated TrEMBL section that indicates the entry has not been manually annotated and reviewed by UniPortKB, it means, it may happen that the sequence slightly differs from genomic sequences. Hence, it can be suggest that NFP is a constant motif in the end of DNA-binding domain. Proline acts as a structural disruptor in the middle of regular secondary structure elements such as alpha helices and beta sheets and is also commonly found in turns, so aids in the formation of beta turns. When proline is located in the end of DNA-binding domain, makes a change in the direction of ERF protein and makes it separates from the DNA that it may help to the function of this protein. It has been previously reported that proline may strongly affect the folding and association of proteins [41]. Hence, it seems proline in the end of AP2/ERF DNA binding domain plays an important role in the conformation of ERF proteins and consequently in their functions.
